# Cardiac Magnetic Resonance in the Assessment of Atrial Cardiomyopathy and Pulmonary Vein Isolation Planning for Atrial Fibrillation

**DOI:** 10.3390/jimaging11050143

**Published:** 2025-05-02

**Authors:** Nicola Pegoraro, Serena Chiarello, Riccardo Bisi, Giuseppe Muscogiuri, Matteo Bertini, Aldo Carnevale, Melchiore Giganti, Alberto Cossu

**Affiliations:** 1 Section of Radiology, Department of Translational Medicine, University of Ferrara, Via L. Ariosto n. 35, 44121 Ferrara, Italy; nicola01.pegoraro@edu.unife.it (N.P.); riccardo01.bisi@edu.unife.it (R.B.); ggm@unife.it (M.G.); 2 Department of Radiology and Laboratory Medicine, University Hospital of Ferrara, 44124 Ferrara, Italy; serena.chiarello@ospfe.it (S.C.); alberto.cossu@unife.it (A.C.); 3 Department of Radiology, ASST Papa Giovanni XXIII Hospital, 24129 Bergamo, Italy; g.muscogiuri@gmail.com; 4 Cardiology Unit, Sant’Anna University Hospital, University of Ferrara, 44124 Ferrara, Italy; matteo.bertini@unife.it

**Keywords:** atrial fibrillation, pulmonary vein isolation, atrial remodeling, cardiac magnetic resonance

## Abstract

Atrial fibrillation (AF) is the most frequently observed type of arrhythmia among adults, and its absolute prevalence is steadily rising in close association with the aging of the population, with its prevalence varying from 2% in the general population to 10–12% among the elderly. The relatively new concepts of ‘atrial cardiomyopathy’ and “AF-related atrial cardiomyopathy”, along with the growing body of knowledge regarding remodeling, function, and tissue characterization, highlight the need for novel approaches to the diagnostic process as well as in the therapeutic guidance and monitoring of atrial arrhythmias. Advanced imaging techniques, particularly cardiac magnetic resonance (CMR) imaging, have emerged as pivotal in the detailed assessment of atrial structure and function. CMR facilitates the precise measurement of left atrial volume and morphology, which are critical predictors of AF recurrence post-intervention. Furthermore, it enables the evaluation of atrial fibrosis using late gadolinium enhancement (LGE), offering a non-invasive method to assess the severity and distribution of fibrotic tissue. The possibility of an accurate CMR pulmonary vein anatomy mapping enhances the precision of pulmonary vein isolation procedures, potentially improving outcomes in AF management. This review underlines the integration of novel diagnostic tools in enhancing the understanding and management of AF, advocating for a shift towards more personalized and effective therapeutic programs.

## 1. Introduction

Atrial fibrillation (AF) is the most frequent arrhythmia among adults, with its prevalence varying from 2% in the general population to 10–12% in elders [1]. Due to the high prevalence of AF, its clinical and public health implications carry significant weight, and when it presents symptomatically, AF is linked to a significant decline in the quality of life. Furthermore, this condition has a significant impact on morbidity and mortality since it is a major risk factor for cardioembolic stroke and heart failure. The management of AF has evolved, particularly with the introduction of catheter procedures targeting pulmonary vein isolation (PVI) [2] and minimally invasive left atrial appendage (LAA) occlusion procedures for embolic stroke prevention. Cardiac magnetic resonance (CMR) and computed tomography angiography (CTA) are currently employed to support the planning of AF ablation and transcatheter LAA occlusion procedures, as well as to assess procedural results and potential complications. CMR has the potential to offer novel insights into atrial cardiomyopathy and the underlying cause of AF by allowing for the investigation of atrial tissue characteristics, morphology, and function. This overview will explore the literature on CMR imaging to assess its current role in supporting AF management.

## 2. Methods

We performed a literature search in Pubmed from 2010 to 2024 using the words atrial fibrillation and cardiac magnetic resonance, as well as left atrium, pulmonary vein isolation, radiofrequency ablation, atrial remodeling, or atrial cardiomyopathy.

The search was limited to English studies, including original studies, reviews, meta-analyses, and case reports.

Studies were selected based on their relevance to the pathophysiological, diagnostic, and prognostic implications of CMR imaging in AF. Articles not primarily focused on AF or without a clear reference to CMR were excluded. The selection process was based on title and abstract screening, followed by full-text assessments for eligibility.

Given the narrative nature of this review, the selection of studies was aimed at capturing key themes and clinically relevant insights rather than providing a comprehensive systematic synthesis.

## 3. Discussion

### 3.1. Atrial Cardiomyopathy

The recently introduced term ‘atrial cardiomyopathy’ (AtCM) identifies the spectrum of the functional and histopathological manifestations of atrial disease. AtCM, by definition, encompasses “any complex of structural, architectural, contractile, or electrophysiological changes affecting the atria with the potential to produce clinically relevant manifestations” [3]. AtCM can be categorized by various etiologies and triggers. For example, some forms primarily involve cardiomyocyte dysfunction due to genetic abnormalities, while others, like fibrotic AtCM, are closely associated with AF [4]. AtCM is characterized by structural alterations such as myocyte hypertrophy, fatty infiltration, and notably left atrial (LA) dilatation and fibrosis [5]. Atrial cardiomyopathy linked to AF (AF-related atrial remodeling or cardiomyopathy) involves a combination of atrial fibrosis and myocyte degeneration [4], with atrial fibrosis being more prominent in people with persistent AF than in those with paroxysmal AF [6]. Initially interfering with electrical conduction, the consequent ischemia, fibroblast proliferation, fibrosis, and extracellular matrix protein accumulation in the myocardial interstitium contribute to structural atrial remodeling and enlargement, which eventually magnifies the AF phenomenon. This results in a self-perpetuating cycle where collagen deposition due to cardiac injury and electrical disturbances further promotes arrhythmia: “AF generates AF” [7]. Subsequent LA dilatation and contractility changes, along with endothelial dysfunction and inflammation, significantly contribute to the risk of thromboembolism in AF. Numerous acquired conditions, including diabetes, obstructive sleep apnea syndrome (OSAS), and obesity, as well as genetic disorders such as atrial natriuretic peptide (NAPP) mutations and muscular dystrophies, are linked to structural changes in the atrial wall and conduction system, which ultimately may eventually result in AF [3]. Additionally, complex disorders such as iron overload in thalassemia syndromes and atrial amyloidosis are more frequently associated with the occurrence of AF [8,9,10]. However, it is important to note that not all the atrial abnormalities seen in individuals with AF are permanent, and it is long known that structural reverse remodeling might occur after successful PVI and sinus rhythm restoration. Specifically, it has been shown that after ablation, atrial volumes and indices of function may improve alongside ventricular diastolic function [11].

### 3.2. CMR in AF-Related Atrial Cardiomyopathy

#### 3.2.1. Left Atrial Volume and Morphology

**Figure 1:**
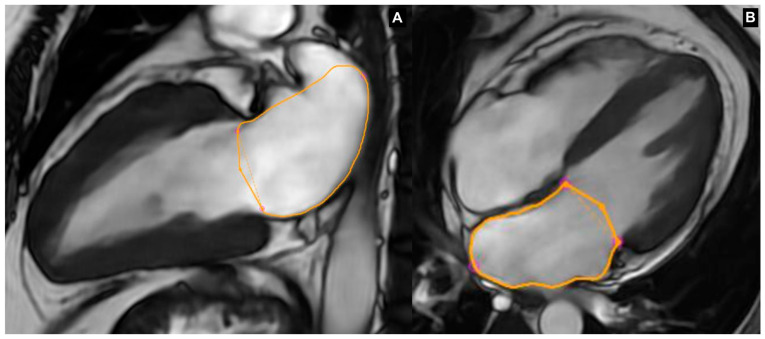
Biplane area–length method approach for quantifying LAV using CMR imaging. (**A**) SSFP image LAX 2CH view; (**B**) SSFP image LAX 4CH view.

Structural changes in the atrium related to AF, such as hypertrophy and electrical remodeling, can lead to myocyte degeneration and interstitial fibrosis, ultimately causing LA dilatation [12]. CMR provides a comprehensive characterization of LA anatomy and volumes, which may facilitate interventional procedures. LA volume (LAV) and LAV indexed (LAVI) are of the most important prognostic predictors and markers of atrial remodeling in AF [13,14]. A clear reciprocal relationship exists between AF and LA enlargement, as they are both causes and consequences of one another [15]. As evidence of progressive AF-related atrial remodeling, patients with persistent AF have been shown to exhibit higher LAVI values than patients with paroxysmal AF and healthy controls [16,17]. The degree of LA remodeling, which is assessed by measuring diameters and volumes, has been suggested as a limiting factor for the effectiveness of PVI procedures in AF. LA dilation serves as a prognostic factor for the recurrence of AF after PVI. After successful AF ablation, a structural reverse remodeling process ensues, resulting in a reduction in LA size [18]. A recent meta-analysis showed that patients who had AF recurrence after radiofrequency ablation (RFA) had higher mean LAV and LAVI [19]. Considering the predictive value of atrial dilatation in terms of both cardiovascular event risk and the likelihood of AF recurrence following treatment, accurately measuring LAV/LAVI is essential for the stratification of patients with AF [20]. The high spatial resolution, excellent atrial endocardial border definition, and capacity for unrestricted dynamic and multiplanar imaging of CMR, particularly SSFP techniques, provide numerous inherent advantages for the assessment of LAV. The biplane area–length method is a widely used approach for quantifying LAV using CMR imaging (Figure 1) [21]. The procedure entails defining the endocardial borders of the LA in the apical four-chamber (A4C) and apical two-chamber (A2C) views. Subsequently, the length of the LA is measured in both the A4C and A2C views, commencing from the center of the mitral annulus to the inner edge of the farthest extent of the traced superior LA wall, approximately at its midpoint. Different cut-offs, normality ranges, and grading systems for LAV and LAVI in adults have been proposed over time for CMR, although the most accepted was proposed by Petersen et al. in 2019 [22]. Also the cut-offs of LAV and LAVI predictors of AF recurrence after PVI procedures have also been proposed over time [23].

Morphological changes in the LA geometry could potentially affect its function and increase the risk of developing arrhythmias like AF, though this connection has not been thoroughly examined. Recent research highlights the significance of LA geometry in the persistence of AF and the risk of recurrence following AF ablation [24,25]. Specifically, alterations in LA sphericity, shifting from a discoid to a more spherical shape during remodeling, might be predictive of AF recurrence [26].

Although the contribution of the left atrial appendage (LAA) to thromboembolic risk is well known, its role in the genesis and recurrence of AF following transcatheter treatment is not fully understood. Nonetheless, some studies have linked the recurrence of AF after PVI to the complex shape and enlargement of the LAA. Particularly, increased LAA volume appears to be associated with the recurrence of arrhythmia [27]. CMR, either through direct assessments or by using dedicated software, enables precise volumetric and geometric assessments of both the LA and LAA.

#### 3.2.2. Left Atrial Function

**Figure 2:**
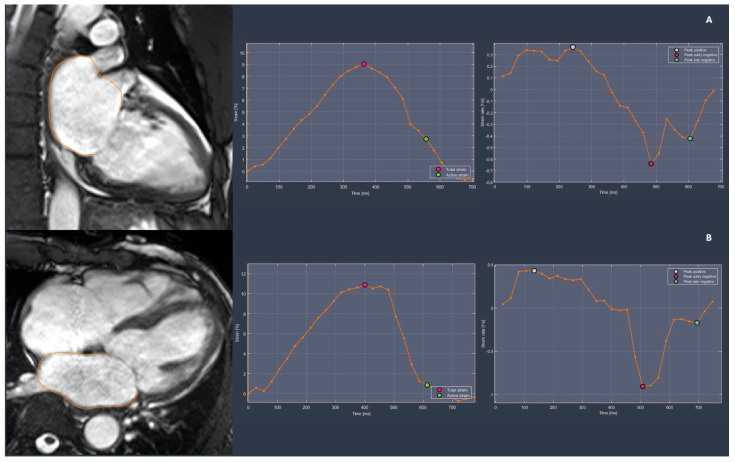
CMR feature tracking method for the assessment of left atrial (LA) strain on steady-state free precession (SSFP) images. LA contouring and strain calculation on the LAX 2CH view (**A**) and on the LAX 4CH view (**B**). Image analysis was done using the freely available software Segment v4.1.0.1 R14284b (Medviso, segment.heiberg.se) [32].

The assessment of LA function through CMR has ascended to the status of the gold standard imaging modality. There are several advantages that CMR has over echocardiography, including a wider field of view, high repeatability, and low intra- and inter-observer variation, especially when applied to the functional assessment of heart chambers [28,29]. The LA plays a critical role in regulating the filling of the left ventricle (LV). During systole, the LA acts as a reservoir for the PV, serves as a conduit for blood flow from the PV to the LV during early diastole, and functions as a pump to enhance LV filling in late diastole [30]. Myocardial strain quantifies the degree of longitudinal deformation in a segment and can be determined by measuring changes in the dimensions of the LA contours within the image plane (Figure 2) [31]. Compared to healthy subjects, patients with AF experience a variable degree of reduction in LA strain function. Patients with persistent AF, compared to those with paroxysmal AF and healthy controls, tend to exhibit lower values of peak longitudinal atrial strain and LA passive emptying fraction, which are representative of reservoir and conduit phase functions, respectively. Additionally, the absolute values of LA systolic, early diastolic, and late diastolic strain rates, representing reservoir, conduit, and LA booster pump functions, are reported to be significantly lower in patients with persistent AF [16]. This finding, evaluated through semi-automatic CMR feature tracking analysis on steady-state free precession (SSFP) images, supports the hypothesis of progressive atrial function loss over time.

Atrial wall fibrosis, a known hallmark of atrial remodeling in AF, is directly associated with atrial strain, with a negative correlation observed between the proportion of LA fibrosis identified by CMR late gadolinium enhancement (LGE) and the atrial strain itself [33]. Furthermore, acute LA disfunction, which is assessed via reduced peak longitudinal atrial strain (PLAS) after PVI, can predict arrhythmia recurrence [34].

A recent study revealed that LA passive function, assessed through CMR strain imaging, is significantly reduced in AF patients with a spherical remodeling of the LA.

LA strain also correlates with atrial stiffness, which is closely related to left ventricular (LV) diastolic function. LA dysfunction is directly linked to the progression of heart failure (HF) with preserved ejection fraction (HFpEF). In fact, LA stiffness has been correlated with an increased risk of all-cause mortality and hospitalization due to HF [35]. Patients with chronic AF exhibit higher stiffness indices, which are associated with the recurrence of AF following PVI, thereby increasing the risk of HFpEF development [36]. In this regard, a new meta-analysis suggests that a non-invasive evaluation of LA stiffness before catheter ablation could serve as a screening tool to identify or monitor patients at an increased risk of recurring AF post-ablation [37]. Unfortunately, stiffness index measurements remain primarily research oriented.

While measurements of left atrial (LA) strain and volume are highly prognostic, inconsistencies arising from differences in atrial measurement techniques and variability in feature tracking analysis complicate the development of standardized diagnostic and prognostic thresholds. The integration of artificial intelligence and machine learning holds promise for enhancing image acquisition, processing, and interpretation, potentially enabling the automated assessment of these fundamental parameters [38].

In AF, structural and electrical remodeling leads to a reduction in LA ejection fraction (LAEF), and baseline values assessed by CMR can predict arrhythmia recurrence [34]. It has also been demonstrated that patients who maintain sinus rhythm after ablation have significantly higher LAEF and left atrial stroke volume (LASV) compared to baseline. In contrast, subjects with recurrent AF do not show such improvements [39]. Furthermore, a recent study indicated that a decrease in LAEF may accurately reflect increased filling pressures associated with chronic diastolic dysfunction, which can occur even before LA remodeling in HFpEF [40].

A recent but established application of phase-contrast CMR is three-directional velocity encoding (4D flow), which allows for the comprehensive in vivo assessment of cardiovascular flow velocities and the quantification of LA blood flow, including stasis fraction. One of the most consistent 4D flow biomarkers in the evaluation of AF is LA peak velocity, which is potentially correlated to the risk of thrombus formation and embolic events [41].

A recent study showed that patients with paroxysmal AF had significantly reduced mean and peak LA flow velocities throughout the entire cardiac cycle and a larger stasis fraction than controls, even in sinus rhythm moments [42]. Those data even show how 4D flow CMR can be a useful tool to better quantify risk assessments in patients with AF.

#### 3.2.3. Left Atrial Tissue Characterization

**Figure 3:**
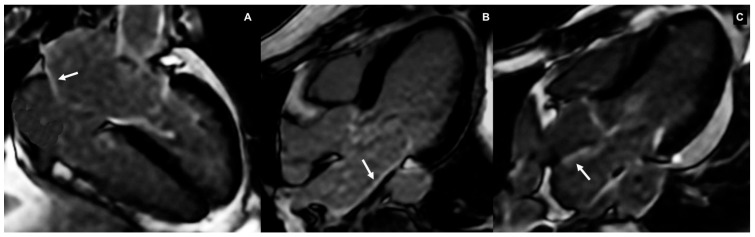
IRGE 2D late gadolinium enhancement (LGE) imaging. Left atrial wall LGE (arrows) on the LAX 4CH (**A**) and 3CH (**B**,**C**) views, respectively.

The application of late gadolinium enhancement (LGE) CMR imaging marks a significant advancement in the precise evaluation of myocardial fibrosis, particularly in the context of AF. CMR’s unparalleled tissue characterization ability aids in the identification of fibrotic or scarred areas within the LA and may provide valuable insights into the substrate of arrhythmogenic foci. Several studies have shown that in patients with AF, areas of LA wall LGE correspond to fibrotic tissue in biopsies (Figure 3). Thus, LGE serves as a powerful, non-invasive tool for assessing atrial fibrosis, providing detailed spatial information about the extent and distribution of fibrotic lesions [43]. LGE imaging is acquired using ECG-gated inversion recovery sequences obtained after injections of gadolinium-based contrast agents, enabling the visualization of areas with increased delayed tissue enhancement. Currently, atrial fibrosis is quantified using a software that employs a threshold-based algorithm. This algorithm considers the degree and intensity of enhancement to measure fibrosis within the LA wall after the epicardial and endocardial margins are manually contoured [44].

A widely used approach for classifying the extent of LA fibrosis is the Utah classification system, which distinguishes four stages of severity based on the amount of left atrial LGE. Although two versions of the system are mentioned in the literature, the most commonly utilized version considers four stages: Stage I—fibrosis extends to less than 10% of the atrial wall; Stage II—between 10% and 20%; Stage III—between 20% and 30%; and Stage IV—more than 30% [45]. This method assists in risk stratification, as the presence and extent of LA fibrosis prior to PVI have been linked to a higher likelihood of post-procedural AF recurrence. Specifically, a correlation has been demonstrated between the Utah stage and ablation outcomes: as fibrosis progresses, the risk of AF recurrence increases, exhibiting varying hazard ratios [46,47]. Patients with a severe fibrotic stage evident on pre-procedural CMR tend to experience a significantly higher rate of arrhythmia recurrence. Therefore, the extent of LGE prior to PVI can be considered an independent predictor of interventional failure and AF recurrence [43]. Moreover, it was shown that patients who remained AF-free after repeated PVI procedures exhibited a decrease in the overall LA LGE. In contrast, the mean scarring thickness did not change in patients who experienced AF recurrence [48]. The development of fibrosis around the PV ostia following isolation is considered a positive outcome, indicating effective intervention. The primary goal of ablation is to isolate and disrupt the abnormal electrical pathways causing AF. The resulting fibrotic response, which encapsulates the area, serves as a beneficial electrical insulator. This barrier helps prevent the spread of faulty electrical signals and reduces the likelihood of AF recurrence.

An excess of epicardial adipose tissue has been associated with atrial AF, indicating that fat may play a significant role in the development of AF arrhythmias through local mechanisms [49]. Using CMR imaging, it has also been shown that there is a significant correlation between epicardial adipose tissue and AF severity and poorer outcomes after AF ablation [50]. Additionally, epicardial fat levels were significantly higher in patients with persistent AF who experienced post-ablation recurrence compared to those who did not [51].

Malagù et al. [52] found that in a contemporary cohort of patients with transfusion-dependent thalassemia, who were well treated with regular chelation therapy, the prevalence of AF was unrelated to iron overload but independently associated with epicardial adipose tissue (EAT).

A relationship has been observed between interatrial septum thickness and intra-atrial fat, indicating that septum thickness could serve as a marker for adipose tissue infiltration in the atria, which is associated with the presence of supraventricular arrhythmias [53].

#### 3.2.4. Left Ventricular Functional and Structural Assessment in AF

**Figure 4:**
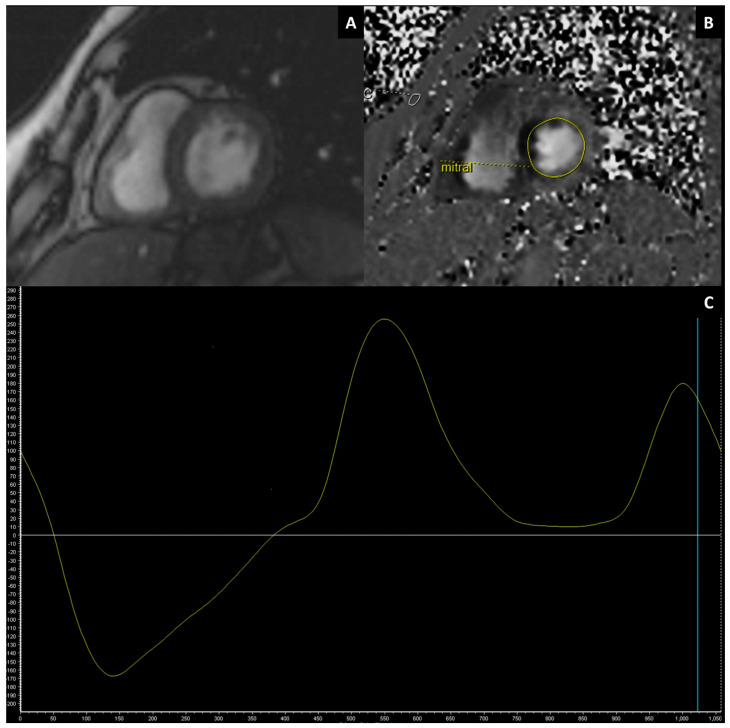
CMR velocity-encoded phase contrast imaging in the evaluation of diastolic left ventricular (LV) function. Trans-mitral SAX magnitude (**A**) and phase contrast (**B**) images and a relative velocity curve (**C**).

It is now well established that AF may affect LV function, causing abnormal diastolic filling and variable degrees of systolic abnormality. It is also clear that a bidirectional cause–effect relationship exists between both LV and LA dysfunction and remodeling. The diagnostic importance of imaging-based diastolic LV function assessments can also be predictive for the future onset of AF, as seen in a recent study that analyzed the LV strain relaxation index through a CMR evaluation in an asymptomatic multi-ethnic population [54]. Although transthoracic echocardiography (TTE) remains the preferred method in the non-invasive evaluation of LV diastolic function, numerous studies have explored the use of CMR-derived diastolic parameters, often demonstrating their non-inferiority. CMR can accurately and reproducibly derive LV diastolic function parameters using velocity-encoded phase contrast imaging. This method measures the trans-mitral (Figure 4) and pulmonary venous flow, employing morphological and quantitative parameters similar to those obtained through TEE [55,56]. Comparable agreement with TTE has been reported for CMR-derived LV volume–time curves [57]. Additionally, 2D and 3D tracking techniques have been explored and proposed for assessing mitral annulus motion, serving as a surrogate for exercise tolerance testing (ETT) in the assessment of left ventricular diastolic function [58]. Recently, Garg et al. proposed an equation for estimating pulmonary capillary wedge pressure (PCWP) based on CMR measurements of LAV and LV mass [59]. PCWP is widely used as a surrogate for LV filling pressure, which is considered the gold standard for definitively diagnosing heart failure with preserved ejection fraction (HFpEF) [60]. Using right heart catheterization as the reference, CMR-derived PCWP has been demonstrated to be significantly superior to TTE in classifying patients with normal or increased LV pressure.

Reduced LV relaxation, a hallmark of HFpEF, is hypothesized to accelerate atrial fibrosis in response to pressure and volume overload [61]. In individuals with a pattern of restrictive LV filling, a recent study reveals more extensive atrial LGE, which relates to an increased risk of arrhythmia relapse after PVI [62].

Not only is diastolic LV function influenced by AF, but several studies have also shown that LV systolic function, as expressed by the LV ejection fraction (LVEF), can be significantly improved in patients with AF when sinus rhythm is restored after PVI [63,64]. CMR is, as already mentioned, the diagnostic gold standard for computing LV volumes and ejection fraction, as it is able to detect changes in systolic function with great accuracy.

Patients undergoing CMR prior to PVI for AF exhibited a high prevalence of unexpected LV scars, with the non-ischemic pattern of LGE being more common [65]. Recent findings also indicate that the presence of LV LGE, assessed by CMR in patients with AF, independently predicts a threefold increase in the risk of major adverse cardiac and cerebrovascular events [66]. The extent of myocardial fibrosis, observable in a remodeled LV in this setting and detectable via LGE imaging, can be considered a significant prognostic factor before the procedure. This factor is crucial for predicting improvements in LV function and the recurrence of AF [63,67].

### 3.3. Pulmonary Vein Isolation (PVI)

PVI is a catheter-based intervention predominantly employed for the management of AF, particularly in cases where pharmacological treatments fail or are contraindicated. Using electrical and anatomical mapping, the procedure involves the precise placement of a catheter within the LA, specifically targeting the antral regions of the PV, which are well-documented sources of ectopic beats initiating and perpetuating AF. Various ablation techniques employing distinct technologies are currently available: through the application of radiofrequency energy, cryoablation or the newest electroporation system, circumferential scars are created around the pulmonary vein ostia [68,69,70,71]. These lesions act to electrically isolate the PVs from the atrial myocardium, thereby preventing the propagation of arrhythmogenic triggers into the atrial tissue. The efficacy of PVI ablation in maintaining sinus rhythm has been substantiated by numerous studies, making it a cornerstone of interventional electrophysiology for AF management [72,73].

### 3.4. CMR in the Planning of PVI

#### Pulmonary Vein Mapping

A normal anatomy configuration of pulmonary veins (PVs), in about 57–82% of the population [74], involves the presence of four pulmonary veins, with two, respectively, for each side independently entering the LA. Anatomic variants are crucial to identify during pre-procedural imaging to ensure accurate mapping and vein isolation. Common anatomical anomalies encompass variations in number or abnormal connections between the PV and the LA. The most frequent variant is the detection of a supernumerary vein, with the most common variation being the presence of a right middle PV (9.0–26.6% of patients) [75]. A “left top” or a “right top” PV can also present less commonly as accessory veins. The most frequent left-side variant reported is the presence of a common drainage trunk, in which PVs of the same side join before entering the LA through the same ostium. Several ostial, branching, and drainage patterns have been documented, with numerous efforts made to classify and categorize them [75]. Also, partial or total anomalous pulmonary venous return can occur. In those cases, a variable number of PVs drain into a systemic vein, determining a left-to-right shunt.

The size of the PV and ostia and their distance from the atrial wall can be assessed with either diameter measurements or measurements of the cross-sectional area. The purpose of pre-procedural imaging is to shorten the procedure’s duration and assess the PV’s anatomy in order to detect anatomical variants (all PVs must be isolated) or vessel stenosis from previous ablation.

**Figure 5:**
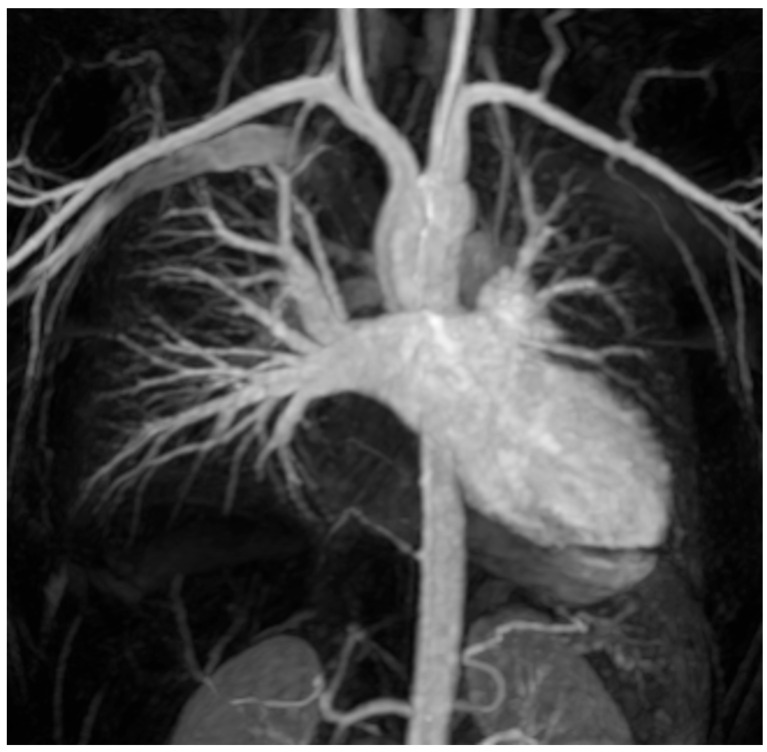
Time-resolved contrast-enhanced MR angiography of the PV for pulmonary vein (PV) Mapping. MIP coronal view of the LV, LA, and PV.

**Figure 6:**
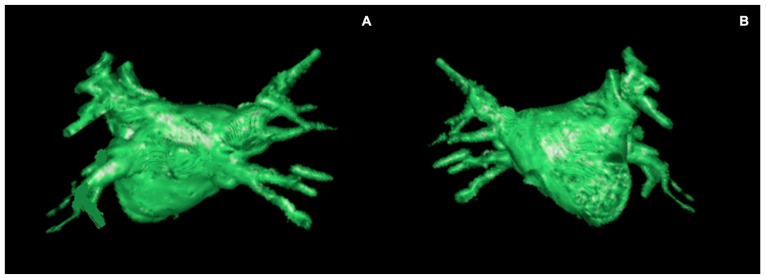
Three-dimensional volume rendering reconstruction of the LA from non-contrast-enhanced three-dimensional (3D) navigator-gated SSFP. (**A**) Posterior view; (**B**) anterior view.

Despite ECG-gated CTA remaining the standard for atrial anatomy evaluation, CMR imaging in pre-ablation pulmonary vein mapping represents a promising advancement in the complex landscape of PVI, offering a non-invasive and comprehensive assessment of PV anatomy [76]. Both conventional contrast-enhanced MR angiography (CE-MRA) and time-resolved contrast MR angiography of the PV can produce contrast-enhanced (CE) PV mapping, with the latter being particularly advantageous, enabling the acquisition of pure pulmonary venous phase images despite the rapid arterio-venous transit time of the pulmonary circulation (Figure 5) [77]. Alternatively, non-contrast MRA techniques may be used, and numerous sequences have been proposed for PV angiography. In particular, non-contrast-enhanced three-dimensional (3D) navigator-gated SSFP MRA represents the most suitable alternative technique to CE-MRA (Figure 6), resulting in non-inferior or even superior image quality [78,79]. Beyond anatomical considerations, functional MRI techniques such as phase-contrast imaging may contribute to the evaluation of blood flow dynamics within the PV, offering insights into hemodynamic parameters that may influence the success of ablation interventions.

Another challenge in the pre-procedural evaluation for PVI is the exclusion of LAA thrombus, which has always been considered essential for minimizing the risk of thromboembolic complications during the intervention. However, recent data indicate that, in patients who are adequately anticoagulated, routine pre-ablation imaging to rule out LAA thrombus may not always be required [80]. The incidence of LAA thrombus is particularly low among individuals with paroxysmal AF in sinus rhythm, while higher rates are observed in those with persistent AF or elevated CHA2DS2–VASc scores [81]. Reflecting these findings, many centers are now reducing their reliance on pre-ablation imaging, especially for patients who are fully anticoagulated. Transesophageal echocardiography (TOE) remains the gold standard for detecting thrombus in the LA or LAA, although it is invasive and necessitates sedation. CTA provides a reliable and less invasive alternative, but it often requires multiple scans, which results in increased radiation exposure [82]. Although CMR imaging with late gadolinium enhancement and longer inversion time has demonstrated efficacy in detecting thrombus, current clinical guidelines still favor TOE or CTA for this purpose [83,84,85]. This ongoing preference highlights a gap between emerging evidence and established recommendations, emphasizing the need for further validation and consensus before the broader adoption of CMR in clinical practice.

The dynamic nature of pulmonary vein anatomy throughout the cardiac cycle can be effectively captured through SSFP cine imaging, allowing for a real-time assessment of PV motion and potential interactions with adjacent structures. Esophageal thermal injury (ETI) is a relatively frequent complication of atrial radiofrequency ablation, occurring in up to 40% of cases [86,87]. The most dramatic epilog of ETI is the complete necrosis of the esophagus wall, atrio-esophageal fistula formation, and mediastinitis. CMR can be used to identify the esophagus’s position and course, as well as potential risk factors for ETI, such as the angle between the esophagus’s posterior wall and the descending aorta. Studies have demonstrated the high diagnostic accuracy of LGE imaging in detecting and assessing the extent and progression of ETI, where the degree of observed enhancement correlates with the severity of ETI. Non-thermal ablation technology for PVI, such as electroporation, has almost completely eliminated the risk of ETI [73].

This multi-faceted approach empowers the electrophysiologist with a comprehensive understanding of the patient’s vascular anatomy, contributing to the refinement of ablation strategies and ultimately enhancing the safety and efficacy of AF PVI procedures.

### 3.5. CMR-Guided Ablation

A hybrid technique has been proposed in AF ablation procedures, combining fluoroscopic and electro-anatomical imaging with the incorporation of pre-procedural CMR data via fusion imaging, with a demonstrated high congruency between the two parameters [88]. This approach aimed to integrate electro-anatomical mapping of the LA with CMR images to create a hybrid map for catheter navigation. Pre-procedural CMR imaging was theorized to reduce procedural duration, radiation exposure, and complication risks, with the potential for better outcomes than standard electrophysiologically guided procedures [71,89,90]. This potential advantage stemmed from imaging’s capacity to detect low-voltage atrial zones, which appeared to correspond to structural abnormalities detected via LGE imaging. Advanced imaging technologies have been explored in transcatheter cardiovascular treatments, with CMR offering anatomical visibility and real-time imaging capabilities [91]. Despite this, the DECAAF-II trial involving patients with persistent atrial fibrillation (AF) found that MRI-guided fibrosis ablation combined with pulmonary vein isolation (PVI) did not significantly reduce atrial arrhythmia recurrence compared to PVI catheter ablation alone [92].

Interventional cardiovascular magnetic resonance (iCMR) applications were developed to address invasive cardiovascular examinations, leveraging CMR’s tissue characterization and eliminating ionizing radiation. Clinical applications ranged from right heart catheterization to hemodynamic tests in congenital heart disease [93]. Early studies suggested the safety of using real-time MRI to guide AF PVI [94]. Effective two-dimensional multi-plane SSFP imaging accelerates acquisitions while maintaining an excellent contrast between blood and tissue. iCMR can distinguish between operative lesions and pre-existing scars [91,95]. Furthermore, temperature mapping has been recently proposed to assess the effect of RF ablation, quantify the heat dose, and anticipate the region of necrotic tissue [96].

## 4. Conclusions

CMR imaging has emerged as a key tool in the comprehensive assessment of patients with AF. It may offer a “one-stop-shop” solution capable of characterizing structural LA anomalies associated with AF-related cardiomyopathy and potential consequences on LV function. Moreover, CMR facilitates planning for PVI, stratifies AF patients, and may help in assessing the risk of arrhythmia recurrence. However, despite its increasing utilization, CMR remains less accessible in many countries, limiting its potential impact on global health. This accessibility gap underscores the need for broader implementation strategies that can bring this crucial technology to more patients worldwide. In addition to accessibility, a major challenge remains, namely the lack of universal adoption and implementation of existing consensus guidelines for atrial volume measurement, functional analysis, and tissue characterization across different centers. Although several consensus guidelines have been proposed over time, variability persists in clinical practice. Addressing these standardization issues will require ongoing collaborative efforts to harmonize protocols, promote multicenter validation studies, and encourage the consistent application of established recommendations. Advances in artificial intelligence and automated image analysis hold promise for improving reproducibility and standardization in CMR-based atrial assessments. Looking forward, future research will concentrate on identifying the predictors of treatment success and patient quality of life, as well as the complications associated with AF-related atrial cardiomyopathy. In conclusion, while the landscape of AF management is evolving rapidly, driven by technological advancements and deeper clinical insights, significant work remains. The ongoing development of imaging techniques and therapeutic strategies will undoubtedly continue to transform our approach to this prevalent and challenging cardiac condition, aiming for improved patient outcomes and a deeper understanding of the disease pathology.

## Data Availability

No new data were created or analyzed in this study. Data sharing is not applicable to this article.

## Abbreviations

AtCM	atrial cardiomyopathy
AF	atrial fibrillation
CE-MRA	contrast-enhanced MR angiography
CTA	computed tomography angiography
TEE	transthoracic echocardiography
TOE	transesophageal echocardiography
EAT	epicardial adipose tissue
ETI	esophageal thermal injury
LA	left atrium
LAA	left atrial appendage
LAV	left atrial volume
LAVI	left atrial volume indexed
LGE	late gadolinium enhancement
LV	left ventricle
PVI	pulmonary vein isolation
RFA	radiofrequency ablation
