# Correction: Pegoraro et al. Cardiac Magnetic Resonance in the Assessment of Atrial Cardiomyopathy and Pulmonary Vein Isolation Planning for Atrial Fibrillation. *J. Imaging* 2025, *11*, 143

**DOI:** 10.3390/jimaging11070233

**Published:** 2025-07-11

**Authors:** Nicola Pegoraro, Serena Chiarello, Riccardo Bisi, Giuseppe Muscogiuri, Matteo Bertini, Aldo Carnevale, Melchiore Giganti, Alberto Cossu

**Affiliations:** 1 Section of Radiology, Department of Translational Medicine, University of Ferrara, Via L. Ariosto n. 35, 44121 Ferrara, Italy; nicola01.pegoraro@edu.unife.it (N.P.); riccardo01.bisi@edu.unife.it (R.B.); ggm@unife.it (M.G.); 2 Department of Radiology and Laboratory Medicine, University Hospital of Ferrara, 44124 Ferrara, Italy; serena.chiarello@ospfe.it (S.C.); alberto.cossu@unife.it (A.C.); 3 Department of Radiology, ASST Papa Giovanni XXIII Hospital, 24129 Bergamo, Italy; g.muscogiuri@gmail.com; 4 Cardiology Unit, Sant’Anna University Hospital, University of Ferrara, 44124 Ferrara, Italy; matteo.bertini@unife.it

In the original publication [1], References 32 and 52 were not cited. 

32. Heiberg, E.; Sjögren, J.; Ugander, M.; Carlsson, M.; Engblom, H.; Arheden, H. Design and validation of Segment - freely available software for cardiovascular image analysis. *BMC Med. Imaging.* **2010**, *10*, 1–13. https://doi.org/10.1186/1471-2342-10-1.

52. Malagù, M.; Tonet, E.; Orazio, G.; Longo, F.; De Raffele, M.; Sirugo, P.; Capanni, A.; Clò, S.; Berloni, M.L.; Marchini, F.; et al. Association between Epicardial Adipose Tissue and Atrial Fibrillation in Patients with Transfusion-Dependent β-Thalassemia. *J. Clin. Med.* **2024**, *13*, 3471. https://doi.org/10.3390/jcm13123471.

The citations have been inserted in the Figure 2 caption and the fourth paragraph of Section 3.2.3. Left Atrial Tissue Characterization. The corrected elements should read as follows:

**Figure 2.** CMR feature tracking method for the assessment of left atrial (LA) strain on steady-state free precession (SSFP) images. LA contouring and strain calculation on the LAX 2CH view (**A**) and on the LAX 4CH view (**B**). Image analysis was done using the freely available software Segment v4.1.0.1 R14284b (Medviso, segment.heiberg.se) [32].

Malagù et al. [52] found that in a contemporary cohort of patients with transfusion-dependent thalassemia, who were well treated with regular chelation therapy, the prevalence of AF was unrelated to iron overload but independently associated with epicardial adipose tissue (EAT).

With this correction, the order of some references has been adjusted accordingly. The authors state that the scientific conclusions are unaffected. This correction was approved by the Academic Editor. The original publication has also been updated.
